# Influence of low viscosity resin infiltrant on the adhesion of orthodontic attachments

**DOI:** 10.1590/2177-6709.30.5.e2525126.oar

**Published:** 2026-01-09

**Authors:** Bruna Caroline Tomé BARRETO, Gabriela Drago VIDAL, Katherine Judith de Carvalho Macário Presado SILVER, Karla Lorene de França LEITE, Carolina Mara Geraldino MONTEIRO, Matheus Melo PITHON, Margareth Maria Gomes de SOUZA

**Affiliations:** 1 Federal University of Rio de Janeiro, Dentistry Course, Department of Pediatric Dentistry and Orthodontics (Rio de Janeiro/RJ, Brazil).; 2 Private Practice.; 3 State University of Southwest Bahia, Department of Health (Jequié/BA, Brazil).

**Keywords:** Shear strength, Dental white spot, Dental caries, Adhesion, Resistência ao cisalhamento, Mancha branca dentária, Cárie dentária, Adesão

## Abstract

**Introduction:**

The search for materials and techniques that promote better adhesion to enamel previously affected by carious lesions has intensified in orthodontics, especially with the use of infiltrating resins and composites with different characteristics.

**Objective:**

To evaluate the adhesion of orthodontic attachments made with different composites bonded to previously infiltrated enamel.

**Methods:**

Ninety bovine lower incisors were randomly divided into 6 groups (n=15): 1 control Z350 (group C) and 5 experimental: Z350 (group Z), bulk fill - 3M (group FM), bulk fill flow - 3M (group FFM), bulk fill - FGM (group FF), bulk fill flow - FGM (FFF). Each group differed by the type of composite used to attachment. Only the experimental groups were subjected to cariogenic challenge followed by treatment with Icon, a low viscosity resinous infiltrant. After aging, orthodontic attachments were bonded to all specimens (n=90) and a mechanical shear bond strength test was performed with subsequent analysis of the adhesive remnant index (ARI). Data were analyzed using Jamovi software version 2.3 with a significance level of 5%. Descriptive statistics (means and standard deviations), normality test and ANOVA/Tukey for shear variables and Kruskal-Wallis test for ARI were performed.

**Results:**

The control group required greater force to shear the attachment (100N ± 32.6; p= 0.016), while the group that used the Bulk Fill Flow FGM composite required less force (55.6N ± 22.5; p= 0.016).

**Conclusion:**

The presence of resin infiltrant influenced the adhesion of orthodontic attachments, made with different composites. However, it did not interfere with ARI.

## RESUMO

**Introdução:**

A busca por materiais e técnicas que promovam melhor adesão ao esmalte previamente afetado por lesões cariosas tem se intensificado na Ortodontia, especialmente com o uso de resinas infiltrantes e compósitos com diferentes características.

**Objetivo:**

Avaliar a adesão de *attachments* ortodônticos confeccionados com diferentes compósitos em esmalte previamente infiltrado.

**Métodos:**

Noventa incisivos inferiores bovinos foram divididos aleatoriamente em 6 grupos (n=15): 1 controle Z350 (grupo C) e 5 experimentais: Z350 (grupo Z), bulk fill - 3M (grupo FM), bulk fill flow - 3M (grupo FFM), bulk fill - FGM (grupo FF), bulk fill flow - FGM (grupo FFF). Cada grupo diferiu quanto ao tipo de compósito utilizado na confecção dos *attachments*. Somente os grupos experimentais foram submetidos ao desafio cariogênico seguido de tratamento com Icon, um infiltrante resinoso de baixa viscosidade. Após o envelhecimento, *attachments* ortodônticos foram colados em todas as amostras (n=90) e realizado o teste mecânico de resistência ao cisalhamento, seguido da análise do índice de remanescente adesivo (IRA). Os dados foram analisados no software Jamovi versão 2.3, com nível de significância de 5%. Foram aplicadas estatísticas descritivas (médias e desvios-padrão), teste de normalidade e ANOVA/Tukey para as variáveis de cisalhamento e Kruskal-Wallis para o IRA.

**Resultados:**

O grupo controle apresentou maior força necessária para o cisalhamento dos acessórios (100 N ± 32,6; p = 0,016), enquanto o grupo que utilizou o compósito Bulk Fill Flow FGM apresentou menor força (55,6 N ± 22,5; p = 0,016).

**Conclusão:**

A presença do infiltrante resinoso influenciou a adesão dos acessórios ortodônticos confeccionados com diferentes compósitos. Contudo, não interferiu no IRA.

## INTRODUCTION

The use of orthodontic appliances promotes the accumulation of dental biofilm, consequently increasing the risk of enamel demineralization around orthodontic accessories.¹ With the advent of clear aligners, it was initially assumed that these issues would be reduced or even eliminated. However, in nearly all cases, clear aligner therapy involves the bonding of attachments,¹ which, like other orthodontic accessories, may facilitate microbial accumulation.² The selection of restorative material for the fabrication of orthodontic attachments represents a critical step in clinical planning. Conventional composite resins have traditionally been used due to their high wear resistance and ability to maintain structural integrity throughout orthodontic treatment.³ The development of bulk-fill composites has allowed clinicians to apply material in larger increments while achieving clinical outcomes comparable to conventional composites in terms of marginal adaptation, restoration integrity, and longevity.⁴ Bulk-fill flowable composites, with lower viscosity, further facilitate adaptation to the internal walls of the attachment matrix. Despite their greater susceptibility to surface wear, experimental studies have shown that these materials maintain clinically acceptable shear bond strength to enamel, supporting their suitability for orthodontic use.^3^ ^,^ ^5^

Although removable aligner therapy presents several advantages, including safety, aesthetics, and patient comfort, studies assessing its impact on oral health remain limited.^1^ ^,^ ^6^ ^,^ ^7^ A prospective randomized clinical trial reported no significant differences in oral hygiene levels between treatments with clear aligners, self-ligating brackets, and conventional brackets.^2^ ^,^ ^8^ Raghavan et al.⁹ and Al-Mutairi et al.¹⁰ demonstrated that patients treated with both fixed appliances and aligners developed white spot lesions on enamel, indicating that microbial accumulation may occur regardless of the type of orthodontic appliance used. 

In the context of white spot formation associated with removable aligners, it is common for attachments to be bonded to vestibular surfaces previously treated for these lesions. The literature indicates that treatment of white spots with Icon^®^ low-viscosity resin infiltrant does not negatively affect the shear bond strength of orthodontic bonding, and simulated aging of the area does not compromise bracket adhesion.^11^ ^,^ ^12^ Conversely, studies have evaluated the remineralization of white spot lesions and its effect on the bond strength of orthodontic brackets bonded to demineralized enamel.¹³ However, evidence regarding orthodontic attachments remains scarce, highlighting the relevance of this investigation. 

Therefore, the objective of this study was to investigate and compare the adhesion of orthodontic attachments fabricated with different composites bonded to previously infiltrated enamel, aiming to determine which material provides optimal adhesion to tooth enamel. 

## MATERIAL AND METHODS

This in vitro study was approved by the Ethics Committee on the Use of Animals in Research at the Universidade Federal do Rio de Janeiro (CEUA/UFRJ). Prior to the experiment, a sample size calculation was performed¹⁴ based on the mean and standard deviation reported by Vianna et al.¹¹ The calculation indicated the need for 12 specimens per group, considering a statistical power of 80% and a significance level of 0.05. To compensate for potential losses, 25% was added, resulting in 15 bovine incisors per group and a total of 90 specimens. Inclusion criteria required sound enamel surfaces without apparent defects, confirmed under halogen light. 

The 90 bovine lower incisors were randomly allocated into six groups according to the composite used for attachment fabrication (Table 1). The teeth were sectioned into cylindrical specimens measuring 6 mm in diameter. The buccal surfaces of the crowns were polished with water-cooled sandpaper using a metallographic polisher (APL4, Arotec, Cotia/SP, Brazil) with sequential grits (#400, #600, and #1200). 

**Table 1:** Characterization of the sample when subjected to the demineralization and remineralization process.

Groups	Composition	Manufacturer (city, state, country)
C	Composite Z350	3M ESPE, St. Paul, MN, EUA
Z	Composite Z350	3M ESPE, St. Paul, MN, EUA
FM	Composite Bulk Fill	3M ESPE, St. Paul, MN, EUA
FFM	Composite Bulk Fill Flow	3M ESPE, St. Paul, MN, EUA
FF	Composite Bulk Fill	FGM, Joinville, SC, Brazil
FFF	Composite Bulk Fill Flow	FGM, Joinville, SC, Brazil

Control group (**C**) and experimental groups (**Z** - 3M Z350 resin, **FM** - 3M bulk fill resin, **FFM** - 3M flowable bulk fill resin, **FF** - FGM bulk fill resin and **FFF** - FGM flowable bulk fill resin).

To induce white spot lesions, specimens from the experimental groups were subjected to a cariogenic challenge, while the control group was not. Artificial saliva, BHI broth, and 2% sucrose were employed for this purpose. Artificial saliva was prepared at the School of Pharmacy (Universidade Federal do Rio de Janeiro, Brazil) with the following composition per liter: 674 mg NaCl, 116.8 mg CaCl₂, 8 g carboxymethylcellulose, 40.8 mg MgCl₂, 960 mg KCl, 1000 mg methylparaben, 24 g liquid sorbitol, 964.9 mL distilled water, and 274 mg monobasic potassium phosphate. Microorganisms were inoculated in 25 mL BHI broth for the growth of *Streptococcus mutans* (ATCC 25175), *Lactobacillus casei* (ATCC 393), and *Candida albicans* (ATCC 90028). For each specimen, 5 mL of medium (BHI + 2% sucrose + microorganism) was applied and incubated for 48 h. After incubation, superficial biofilm layers were removed with 2 mL MilliQ water to allow assessment of white spot lesions. 

Lesion detection was performed according to the International Caries Detection and Assessment System (ICDAS), using the following criteria: Code 0 = sound enamel surface; Code 1 = white/brown opacity visible on dry enamel; Code 2 = white/brown opacity visible on wet enamel. A positive result was defined as the presence of a white spot lesion on enamel, under either dry (Code 1) or wet (Code 2) conditions. For reproducibility analysis, 30% of the sample was re-examined after two weeks by two independent evaluators (B.C.T.B. and G.D.V.), and intra- and inter-rater agreement was calculated using the intraclass correlation coefficient (ICC).

Once lesions were confirmed, infiltrant resin (Icon^®^, DMG, São Paulo, Brazil) was applied following the manufacturer’s instructions. Experimental groups were subsequently stored in artificial saliva at 37 °C for 56 h, with renewal every 8 h.^15^ ^,^ ^16^ According to the manufacturer, Icon^®^ infiltrates the microporosities of white spot lesions, sealing diffusion pathways of cariogenic acids and thereby arresting lesion progression. 

Aging of the specimens was performed using ultraviolet irradiation with a tungsten filament lamp in a mercury vapor atmosphere,^11^ ^,^ ^17^ following ADA Standard no. 27. Conditions included a wavelength of 365nm, temperature of 45 °C, and 65% relative humidity in a darkroom (Model SL-204, Solab, Piracicaba, Brazil). Exposure lasted 72 h, corresponding to 15 years of natural aging (ISO 3336-19f7).¹⁸ 

**Figure 1:**
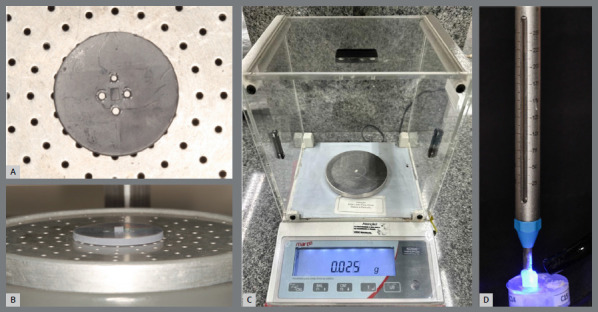
Printed model positioned in approximate view **(A)** and oblique view **(B)**; Template with resin included weighing 0.025 grams **(C)**; Tensiometer exerting a force of 2.94 N on the polymerization of the composite **(D)**.

Orthodontic attachments were standardized to a rectangular shape (3 mm height × 2 mm width × 2 mm depth). A virtual model of the attachments was designed using Meshmixer™ Software (v.3.5.474, Autodesk™, Inc., Delaware, USA) and 3D-printed (Form 2, Formlabs, Massachusetts, USA) with SLA resin (ClickDental, Novo Hamburgo, RS, Brazil). Templates were fabricated using a vacuum laminator (PlastVac P7, Bio Art, São Carlos, SP, Brazil) with a 0.3 mm crystal-type thermoplastic plate (Bio Art, São Carlos, SP, Brazil). One template was produced per specimen (Fig. 1). 

Enamel conditioning was performed with 37% phosphoric acid (FGM, Joinville, SC, Brazil) for 20 s, followed by rinsing and drying for the same duration. Adhesive systems were applied according to the composite group: Single Bond Universal (3M, São Paulo, SP, Brazil) for Filtek™ Z350, Filtek™ Bulk Fill, and Filtek™ Bulk Fill Flow; and Ambar APS (FGM, Joinville/SC, Brazil) for Opus Bulk Fill and Opus Bulk Fill Flow. Adhesive was applied to the bonding area and air-thinned to evaporate solvents.

Composite resin was inserted into each template and standardized by weight (0.025 g) using a precision scale. Pressure was applied with a tensiometer at 2.94 N to ensure uniform placement. Light curing was performed for 40 s with an LED unit at 1100 mW/cm², regularly monitored with a radiometer (Demetron, Danbury, CT, USA). The control group attachments were bonded directly to sound enamel. 

**Figure 2:**
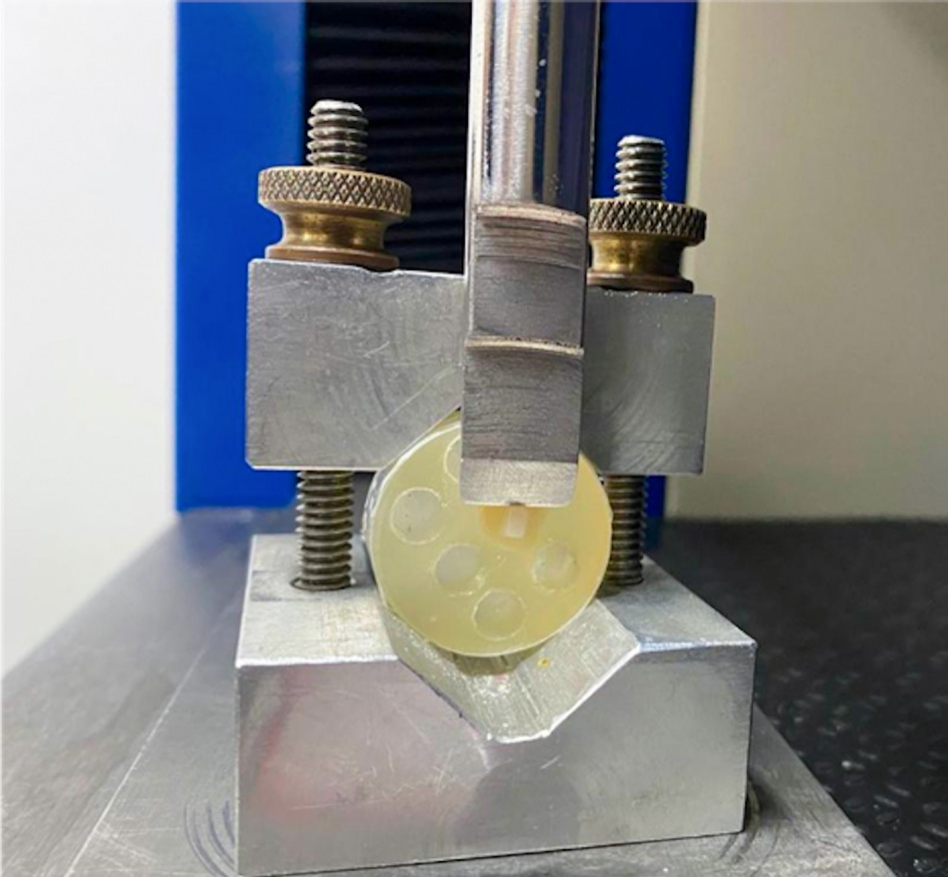
Mechanical shear bond strength test.

Shear bond strength testing was performed in a universal testing machine (EMIC DL10.000, São José dos Pinhais, Brazil) with a crosshead speed of 0.5 mm/min and a 0,5N load cell. A chisel-shaped active tip was positioned at the lateral aspect of each attachment (3 mm), and failure load was recorded in Newtons (N) at the point of debonding (Fig. 2). 

Post-test evaluation of the enamel surface was carried out under a stereomicroscope (OptIcam, São Paulo, Brazil) at 20× magnification. The adhesive remnant index (ARI) was scored as follows: 0 = no composite remaining on enamel; 1 = 50% of composite remaining; 3 = all composite remaining.¹⁹ 

Repeated measurements from 30% of the sample, over a two-week interval, were randomly selected, measured by two raters (B.C.T.B. and G.D.V.) and correlated to determine inter and intra-rater agreement using the ICC. 

Descriptive statistics (mean and standard deviation) were used to report shear bond strength values for each group. Assumptions of normality and homogeneity of variance were verified using the Shapiro-Wilk and Levene tests, respectively. Differences among groups were analyzed with one-way ANOVA followed by Tukey’s post hoc test. ARI scores were analyzed using the Kruskal-Wallis test. Statistical analyses were conducted in JAMOVI software (v.2.3), with significance set at 5%. 

## RESULTS

Visual assessment of white spot lesions demonstrated high reproducibility, with inter-examiner reliability of 0.919 and intra-examiner reliability of 0.825 and 0.880 for each examiner, respectively, indicating almost perfect agreement across all comparisons.²⁰ All groups exhibited the formation of white or brown spots on wet enamel in at least 75% of specimens, while the remaining specimens presented white spots on dry enamel. No specimen remained free of lesions following bacterial adhesion. 

The mean values and standard deviations of the shear bond strength test are presented in Table 2. The control group required the highest shear force (100 ± 32.6N), whereas the FFF group showed the lowest values (55.6 ± 22.5N; 9.2 ± 3.7 MPa). A statistically significant difference was observed between these groups (p = 0.016; p = 0.020). 

**Table 2:** Mean and standard deviation of force (N) found in the shear test and p value from ANOVA’s test.

Group	Force (N)
C	100 (32.6)^a^
Z	74.1 (32.1)^ab^
FM	90.3 (41.5)^ab^
FFM	78.6 (39.9)^ab^
FF	76.4 (32.6)^ab^
FFF	55.6 (22.5)^b^
p value	0.016

Different letters show statistical significance.

Adhesive remnant index (ARI) classification also demonstrated high reproducibility, with inter-examiner reliability of 0.911 and intra-examiner reliability of 0.907 and 0.943, confirming almost perfect agreement.²⁰ The distribution of ARI scores is shown in Table 3. The Kruskal-Wallis test revealed a statistically significant difference among groups (p = 0.020). The control group presented the highest proportion of specimens with score 3 (all composite remaining on enamel), while the FF group exhibited a higher number of specimens with score 1 (<50% of composite remaining). Both FFM and FFF groups (flowable composites) showed the majority of their specimens classified as score 1.

**Table 3:** ARI scores by group using the Kruskal-Wallis test.

	0	1	2	3
C^a^	0	3	5	7
Z^ab^	0	3	7	5
FM^a^	0	5	5	5
FFM^a^	0	8	3	4
FF^b^	0	10	5	0
FFF^a^	0	8	4	3
p value	0.020	0.020	0.020	0.020

0 = no amount of composite adhered to the enamel; 1 = less than half of the composite adhered to the enamel; 2 = more than half of the composite adhered to the enamel; 3 = all the composite adhered to the enamel^12^. Different letters show statistical significance.

## DISCUSSION

This study aimed to evaluate the possible influence of a low-viscosity resin infiltrant on the shear bond strength of orthodontic attachments. To simulate the development of white spot lesions, specimens were subjected to a cariogenic challenge, in accordance with the literature, which identifies the white spot as the earliest clinical manifestation of carious lesions.^21^ ^-^ ^23^ The appearance of these lesions is known to be directly related to the demineralization-remineralization dynamics of enamel,^24^ ^-^ ^26^ occurring when the balance shifts toward demineralization as a consequence of pH reduction.²⁶ The microorganisms employed in this study (*Streptococcus mutans, Lactobacillus casei,* and *Candida albicans*) were selected based on evidence demonstrating that an acidic environment promotes their proliferation, contributing to enamel and dentin demineralization.²⁷ White spot lesion identification and classification were performed visually, in line with well-established methodologies.²⁸

With regard to the resin infiltrant, the present study confirmed its effectiveness in the management of white spot lesions, in agreement with previous reports.^28^ ^-^ ^31^ According to Tavares et al.,³² this minimally invasive approach represents a promising therapy due to its ability to penetrate and infiltrate the dental substrate, thereby preventing lesion progression by blocking the diffusion of acids and minerals. Moreover, it has been shown to significantly increase enamel surface microhardness,³⁰ an effect consistently supported by other investigations.^29^ ^,^ ^31^ Despite these advantages, the findings of this study indicate that resin infiltration may compromise the adhesion of orthodontic attachments, as lower shear bond strength values were observed in the experimental groups compared with the control, in which the infiltrant was not applied. In contrast, Ozcan et al.¹² reported no reduction in bracket bond strength when Icon^®^ was used, a discrepancy that may be explained by methodological differences: the present study tested composite resin attachments bonded with adhesive systems, whereas Ozcan et al.^12^ employed orthodontic adhesive and metallic brackets. Similarly, Resende et al.³³ found no significant changes in shear bond strength when Transbond XT orthodontic adhesive was evaluated. Furthermore, in the present study, the FFF group exhibited the lowest bond strength values, which may be attributed to the intrinsic characteristics of flowable resins, including their reduced filler content, lower microhardness, and increased susceptibility to wear.^34^ ^,^ ^35^

The flow resin has low bond strength, as found by de Jesus Jesus Tavarez et al.^36^ In their study, the flowable resin presents the lowest values of micro-shear bond strength in association with the Z350 resin. Among other factors, this property can be explained by the low microhardness that affects the bond strength,^37^ in addition to making the flow resin more susceptible to deformation and wear, compromising the stability of the bond.^38^ Although bulk fill composites are not the most obvious types of composites for the application used in this study, they were used in an attempt to offer another option to clinicians. 

Artificial aging was incorporated into the experimental design to simulate the effects of time.³⁹ The decision to include aging in the study is based on previous research that tested bond strength after aging with infiltrating resin.^40^ ^,^ ^41^ Previous studies have shown that artificial aging reduces bond strength, indicating that long-term durability of adhesion may be compromised.^42^ ^-^ ^45^ Moreover, viscosity has been shown to influence material penetration and bond strength.^43^ The aging protocol employed here followed Vianna et al.,¹¹ who simulated a clinical condition in which Icon^®^-treated white spot lesions subsequently received orthodontic bonding. In the present study, a comparable scenario was reproduced with attachments used in aligner therapy, thereby simulating an adult patient beginning treatment years after the development of white spot lesions during childhood or adolescence. This approach reflects the increasing popularity of aligner therapy among adult patients worldwide. 

Regarding shear bond strength of orthodontic brackets, several studies have evaluated the influence of infiltrant resin on adhesion,^12^ ^,^ ^46^ yet no studies to date have focused on orthodontic attachments. It is well established that demineralized enamel reduces bracket bond strength compared with sound enamel.⁴⁷ Some investigations report that the use of Icon^®^ does not alter^13^ ^,^ ^48^ or may even increase bond strength,^13^ ^,^ ^46^ whereas others suggest that infiltration modifies bonding characteristics.^49^ ^-^ ^51^ Ekizer et al.^50^ observed higher bond strength on infiltrated enamel compared with untreated demineralized enamel, suggesting a potential benefit of resin infiltration in compromised surfaces. In the present study, however, greater bond strength was required to debond attachments in the control group (without resin infiltrant) compared with experimental groups. No significant difference was found between the control and Z350 groups, whereas bulk-fill composites (FM and FF) exhibited higher resistance than their flowable counterparts (FFM and FFF). This supports the preferential use of bulk-fill composites over flowable resins for attachment fabrication. 

The results suggest that the inferior performance of the flowable resin, which exhibited greater wear, may be associated with the intrinsic properties of this type of material. An important aspect to consider is the lower microhardness typically observed in flowable composites, a characteristic that may compromise their mechanical resistance under masticatory loads. The reduced inorganic filler content and the higher proportion of organic matrix in these formulations decrease the material’s ability to withstand abrasion and plastic deformation, which partly explains the inferior performance observed in this study. Therefore, the correlation between microhardness and clinical durability should be emphasized, highlighting that the selection of composite resin should take into account not only ease of handling but also its structural limitations.

Regarding ARI, no significant differences were observed between the control and Z350 groups, suggesting that low-viscosity resin infiltration does not substantially affect adhesive remnant distribution when using the same composite material. 

These results have direct implications for aligner biomechanics. Attachments are essential for transmitting forces required for extrusion, rotation, and torque. Compromised adhesion reduces attachment retention, which may lead to force loss and impaired movement predictability. A minimum shear bond strength of 6-8 MPa is considered clinically acceptable to prevent frequent debonding.^52^ In the present study, control and conventional composite groups achieved values above this threshold, while the FFF group approached or fell below it, raising concerns for clinical stability. 

The inferior performance of flowable composites is consistent with previous studies reporting significantly lower SBS compared with conventional adhesives.^53^ Although some investigations suggest that resin infiltrants such as Icon^®^ may not impair or even improve bracket bonding,^54^ our findings indicate a material and protocol dependent influence, with flowable composites being most affected. 

From a clinical perspective, these results emphasize the importance of preferring conventional or bulk-fill composites over flowables for attachments, especially on infiltrated enamel, to ensure long-term retention and effective force transmission. Accurate attachment design and bonding technique further contribute to maintaining aligner biomechanics and minimizing treatment delays.^55^

The present study has several limitations that should be acknowledged. First, the aging protocol adopted (ADA No. 27) did not include thermal cycling or other thermomechanical procedures capable of more accurately reproducing fatigue and hydrolytic degradation in the oral environment. Second, the control group was not subjected to the same storage conditions as the experimental groups, which may have introduced potential bias. Another limitation concerns the use of bovine teeth, which, although presenting morphological characteristics similar to human dental tissues and being routinely employed in bond strength studies,^56^ cannot faithfully reproduce the clinical environment of the oral cavity. Additionally, different adhesive systems were employed according to the manufacturer’s recommendation for each composite resin. While this approach aimed to ensure optimal bonding performance under clinically relevant conditions, it may also have introduced a confounding factor, since variations in adhesive formulation and application protocols can influence shear bond strength. Therefore, the differences observed among composite groups should be interpreted with caution, as they may partially reflect adhesive characteristics rather than only the intrinsic properties of the composite resins. Furthermore, due to the lack of standardized in vitro testing protocols for orthodontic attachments in the literature, we adapted the methodology commonly used for brackets with a chisel-shaped active tip. Although this ensured reproducibility and comparability with previous studies, it does not fully replicate the complex biomechanical forces generated during clear aligner therapy. 

Despite these limitations, the present study contributes original data to the literature by evaluating the adhesion of orthodontic attachments rather than brackets. The methodological rigor applied -including enamel surface standardization and strict control of attachment dimensions and weight- strengthens the reliability of the findings. Clinically, optimal adhesion at the attachment-enamel interface is critical for the longevity of attachments, reducing failures, minimizing emergency appointments, avoiding unnecessary chairside time, and preventing repeated enamel wear due to rebonding procedures. 

## CONCLUSIONS

Bond strength was more efficient on healthy dental surfaces when compared to surfaces that had suffered white spot lesion and treatment with a low viscosity resinous infiltrant. The effect of the infiltrant on adhesion capacity was material-dependent, with a statistically significant reduction observed only for the bulk-fill flowable resin group, while no significant difference was found for the conventional composite compared with the control. And the flowable composites, particularly the FFF group, exhibited inferior performance under the conditions tested in this study, regardless of the use of the infiltrant. 
